# *Myco*Wiki: Functional annotation of the minimal model organism *Mycoplasma pneumoniae*

**DOI:** 10.3389/fmicb.2022.935066

**Published:** 2022-07-25

**Authors:** Christoph Elfmann, Bingyao Zhu, Tiago Pedreira, Ben Hoßbach, Maria Lluch-Senar, Luis Serrano, Jörg Stülke

**Affiliations:** ^1^Department of General Microbiology, Göttingen Center for Molecular Biosciences, Georg-August University Göttingen, Göttingen, Germany; ^2^EMBL/CRG Systems Biology Research Unit, Centre for Genomic Regulation (CRG), Universitat Pompeu Fabra (UPF), Barcelona, Spain

**Keywords:** *Myco*Wiki, genome annotation, essential genes, systems biology, database

## Abstract

The human pathogen *Mycoplasma pneumoniae* is viable independently from host cells or organisms, despite its strongly reduced genome with only about 700 protein-coding genes. The investigation of *M. pneumoniae* can therefore help to obtain general insights concerning the basic requirements for cellular life. Accordingly, *M. pneumoniae* has become a model organism for systems biology in the past decade. To support the investigation of the components of this minimal bacterium, we have generated the database *Myco*Wiki. (http://mycowiki.uni-goettingen.de) *Myco*Wiki organizes data under a relational database and provides access to curated and state-of-the-art information on the genes and proteins of *M. pneumoniae*. Interestingly, *M. pneumoniae* has undergone an evolution that resulted in the limited similarity of many proteins to proteins of model organisms. To facilitate the analysis of the functions of *M. pneumoniae* proteins, we have integrated structure predictions from the AlphaFold Protein Structure Database for most proteins, structural information resulting from *in vivo* cross-linking, and protein-protein interactions based on a global *in vivo* study. *Myco*Wiki is an important tool for the systems and synthetic biology community that will support the comprehensive understanding of a minimal organism and the functional annotation of so far uncharacterized proteins.

## Introduction

Bacteria of the genus *Mycoplasma* are characterized by their strongly reduced genomes that still encode all the functions required for autonomous growth. Bacteria such as *Mycoplasma genitalium* and *Mycoplasma pneumoniae* have genomes of only 480 and 816 kb and encode about 480 and 700 proteins, respectively. These small genomes have put these bacteria into the spotlight of systems and synthetic biology, two recent disciplines in biology that aim for a complete understanding of all processes in a living cell up to mathematic modeling and for the creation of artificial forms of life, respectively.

Starting with global analyses of the metabolism, gene expression, and protein-protein interactions in 2009 (Güell et al., 2009; Kühner et al., 2009; Yus et al., 2009), *M. pneumoniae* has become one of the model organisms of systems biology. Many aspects of its biology such as metabolism, DNA and protein modifications, the micro-proteome, protein degradation, regulatory networks, and gene essentiality have been studied at the global level as well (Schmidl et al., 2010; van Noort et al., 2012; Lluch-Senar et al., 2013, 2015; Wodke et al., 2013; Miravet-Verde et al., 2019; Yus et al., 2019; Burgos et al., 2020; Montero-Blay et al., 2020). The small proteome of *M. pneumoniae* facilitates the investigation of protein function at the global scale as revealed by the first large-scale global *in vivo* study of protein-protein interactions. This analysis resulted in the visualization of important protein complexes and in the identification of functions of so far unknown proteins (O’Reilly et al., 2020).

In addition to its role in systems biology, *M. pneumoniae* is also intensively studied due to its role as a lung pathogen (Meyer Sauteur et al., 2014; Waites et al., 2017; Esposito et al., 2021). Its main virulence determinants are a specific ADP-ribosylating and vacuolating cytotoxin (CARDS, MPN372) (Kannan and Baseman, 2006; Becker et al., 2015), hydrogen peroxide which is produced by glycerol phosphate oxidase (GlpO) as a product of phospholipid and glycerol utilization (Schmidl et al., 2011; Blötz and Stülke, 2017), hydrogen sulfide is produced by the cysteine desulfurase HapE during cysteine degradation (Großhennig et al., 2016), and the immunoglobulin binding protein IbpM helps the bacteria to escape the human immune system (Blötz et al., 2020).

As a minimal pathogen, *M. pneumoniae* might also be useful in fighting disease by delivering therapeutics to the human host or by directly combatting other bacteria (Piñero-Lambea et al., 2015; Garrido et al., 2021). Such applications are favored by the fact that the genetic code used by *M. pneumoniae* is unique, thus preventing horizontal gene transfer, and by the development of methods that allow the construction of attenuated strains by deleting the genes that encode virulence factors. Indeed, this strategy has recently been employed to eliminate biofilms of the harmful and often multiresistant human pathogen *Staphylococcus aureus* (Garrido et al., 2021).

The importance of *M. pneumoniae* as a human pathogen, as a potential therapeutic agent, and its role in systems and synthetic biology suggests that this bacterium will remain the focus of intense research. This requires tools that allow easy access to all available information on the genes and proteins of *M. pneumoniae* and their functional and regulatory interactions. To facilitate the investigation of *M. pneumoniae*, we have developed *Myco*Wiki, a database centered around the genes and proteins of this bacterium. This database shares its framework with the established databases *Subti*Wiki and *Syn*Wiki, which provide functional annotation of *Bacillus subtilis* and the artificial minimal organism *Mycoplasma mycoides* JCVI-syn3A, respectively (Pedreira et al., 2022a,b). *Myco*Wiki presents the available information on the genes and proteins of *M. pneumoniae* in a highly intuitive manner. A particular focus on *Myco*Wiki is the presentation of links and interactions between different genes and proteins, which allows the scientific community to develop novel hypotheses. The information provided in *Myco*Wiki is derived from earlier annotations of the *M. pneumoniae* genome (Dandekar et al., 2000; Wodke et al., 2015) and the published body of knowledge.

## Description of the database

*Myco*Wiki (http://mycowiki.uni-goettingen.de) is built upon the same framework as the aforementioned databases *Subti*Wiki and *Syn*Wiki (Pedreira et al., 2022a,b). As a result, the general organization of data entities and their relations to each other, and the layout of the web pages, are the same. However, some features are exclusive to *Myco*Wiki, such as the representation of cross-linking data combined with protein structures.

The structure of *Myco*Wiki is centered around genes and their products. Most of the information represented in this database is associated with a specific gene/protein, and thus the *Gene* pages are the core part of *Myco*Wiki. They integrate the most data relating to a particular gene, but also connect to separate web pages, for example, pages on certain groups of genes, such as specific functional categories. The *Gene* page also links to *browsers*, which allow exploring some aspects of a gene or the gene product and possible interactions of the encoded protein (such as the *Expression*, *Interaction, and Pathway Browsers*).

## The front page

**FIGURE 1:**
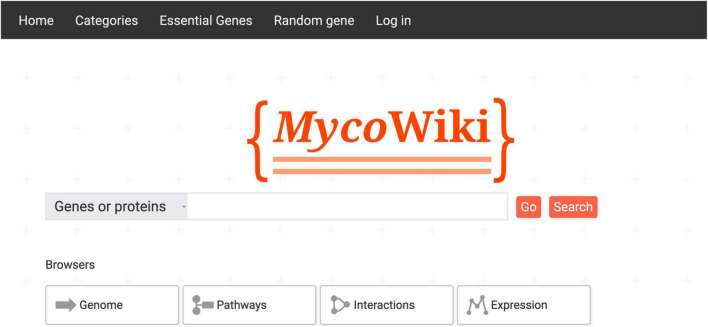
Front page of *Myco*Wiki. The central search bar allows to query genes by name or locus tag, but also facilitates full-text searches. Below, the data browsers are linked for quick access. In the top bar, direct access to an overview of categories and a list of essential genes is provided, and utility links for jumping to a random gene page and for logging in.

The front page of *Myco*Wiki gives access to the *Gene* pages *via* a search bar, which can be used to query genes by unique identifiers (Figure 1). One option is to use a gene’s *name*, usually a mnemonic of three or four letters as it is commonly the case for bacterial genes (such as *eno* for enolase). Genes can also be identified *via* their locus tags, which are largely based on genome re-annotations (Dandekar et al., 2000; Lluch-Senar et al., 2015). For example, *MPN606* is the locus tag for *eno*, and it is guaranteed to never change, even if the mnemonic designation of the gene should be changed. In some cases, a name has not been assigned to a gene yet, so the locus tag is the primary identifier. Aside from these two identifiers, a full-text search of a gene’s data is possible *via* the “Search” button.

**FIGURE 2:**
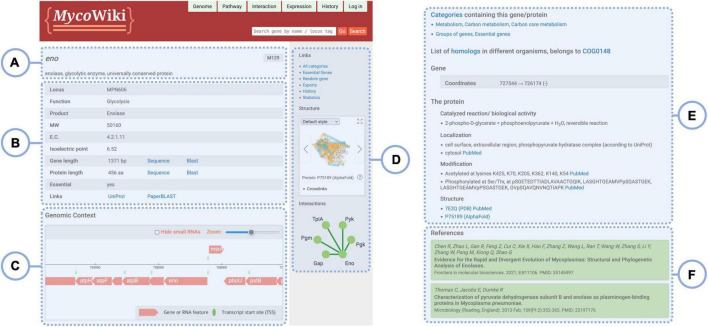
The gene page for *eno*. The general structure of gene pages is the same for all genes, but individual sections and interactive elements may vary due to different information being available. **(A)** Gene name, strain, and description; **(B)** table summarizing basic information on the gene and its product; **(C)** embedded genomic region display; **(D)** sidebar containing helpful links and additional tools, such as the *Structure Viewer*; **(E)** further sections describing various aspects of the gene; and **(F)** list of publications as sources of information.

Moreover, the top bar of the front page gives access to an overview of the functional categories each gene/protein was assigned to, a list of essential genes, and to a random gene page. Finally, it allows the user to log into the database. These links also appear on all gene pages in the right-side bar (Figure 2D, see below). Below the search bar, links to the interactive *Myco*Wiki browsers (see below) are provided.

## The gene pages

In *Myco*Wiki, the *Gene* pages provide access to all data relating to a particular gene. Most of the annotation can be directly viewed on the page, and links to browsers are provided which investigate certain aspects of the gene in more detail. All gene pages share the same basic structure. Figure 2 shows the page for *eno*. The top bar (Figure 2A) contains links to the data browsers, the change history of the page, and a log-in pop-up. It also features the search bar, which has the same functionality as the one presented on the front page. At the top of the main view (Figure 2B), the gene name is indicated as the page title. Below, a short general description is displayed, and the name of the *M. pneumoniae* strain M129 is shown on the side. Next, a table summarizes some basic information about the gene such as the locus tag, function, and sequence information. The latter is accompanied by utility links used to directly BLAST (Sayers et al., 2022) the nucleotide or translated amino acid sequence. This table also features data on the gene’s product, for example, the molecular weight and enzyme commission number of the encoded protein. Further down, an interactive presentation of the genomic region is embedded in the page (Figure 2C), which allows viewing the genomic neighborhood of the gene. It does not feature all of the functionality of the full *Genome Browser*, which can be accessed *via* the top bar, and which will be explained below. On the sidebar (Figure 2D), a group of links provides access to helpful pages. Depending on the gene and the available data, additional interactive elements follow: the *Structure Viewer* shows 3D visualizations of the protein structure and cross-linking data, if available (see below). The *Interaction overview* displays a graph of the protein-protein interactions between the protein and its interaction partners. Proteins are represented by nodes that can be clicked to open the corresponding gene pages. In addition, the edges, which depict interactions, link to relevant publications.

Further down below on the page (Figure 2E), additional sections shed light on various aspects of the gene/protein, such as assigned categories, genomic coordinates, details about the gene product, and other data. At the end of the page, a list of relevant publications is featured (Figure 2F).

### Browsers

*Myco*Wiki and its siblings *Subti*Wiki and *Syn*Wiki feature various *browsers*, which are interactive, graphical displays that allow users to explore certain types of data in an intuitive manner. When they are accessed *via* a gene page’s top bar, the data corresponding to that gene are highlighted. However, data on other genes can be easily loaded in the browsers by the use of search bars, which allows the user to compare and contrast information on multiple genes effortlessly. These search bars are located in the top left corner of any browser. *Myco*Wiki currently features four different browsers.

**FIGURE 3:**
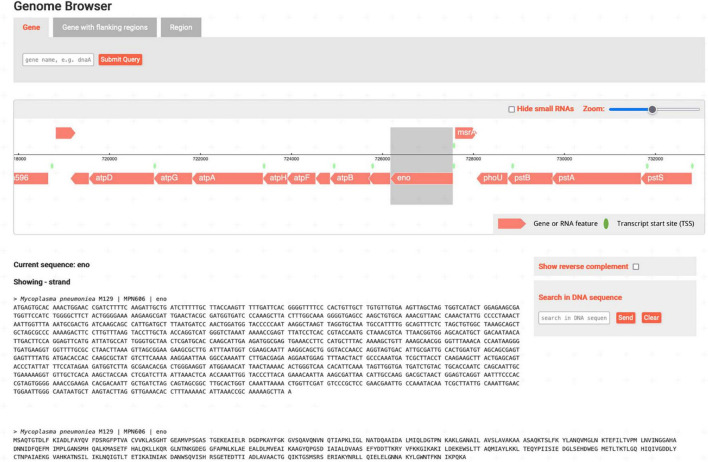
*Genome Browser* page with *eno* selected. The interactive display allows to scroll through the organism’s genome and to load nucleotide and amino acid sequences of genes.

The *Genome Browser* (Figure 3) allows the user to see the immediate neighborhood of the gene and the orientations and lengths of genes, scroll through the genome, and adjust the zoom level. In addition, it includes the display of DNA and protein sequences. Clicking on a gene displays the corresponding sequences below the interactive genome display, where the user can search for substrings and toggle the reverse complement sequence. Flanking regions of genes or freely defined substrings of the genome can be loaded *via* the search bar.

**FIGURE 4:**
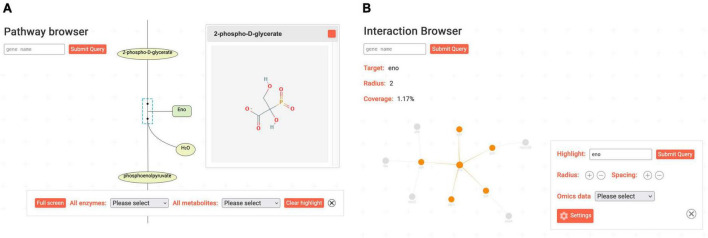
*Interaction* and *Pathway Browser* with Eno highlighted. **(A)** The *Pathway Browser* features a curated map of metabolic reactions. Selected enzymes and metabolites can be highlighted using the toolbar. **(B)** The *Interaction Browser* allows viewing interaction networks of genes. With the toolbar, the size and appearance of the currently viewed network can be adjusted.

The *Pathway Browser* visualizes a curated map of metabolic pathways and related metabolites and enzymes for *M. pneumoniae*. In Figure 4A, the reaction catalyzed by Eno as part of the glycolytic pathway is shown. Clicking on enzymes in the map opens a small pop-up window featuring a basic summary for the corresponding gene/protein. Using the collapsible toolbar, the user can enter a full-screen mode and select enzymes or metabolites to be highlighted.

Protein-protein interactions are important clues to characterize proteins of unknown function. *M. pneumoniae* is the first organism for which a global analysis of the *in vivo* interactome was performed (O’Reilly et al., 2020). The results and the outcome of other more protein-specific studies are displayed in the *Interaction Browser*. In this browser, networks of interacting proteins can be visualized in a dynamic and interactive manner (Figure 4B). Similar to the corresponding interactive element on the gene page sidebar, proteins and their interactions are represented by nodes and edges of a graph. However, the browser display is more flexible: the user can rearrange nodes by dragging them with the cursor, and other visualization options can be adjusted *via* the toolbar. More proteins can be included in the display by increasing the *radius*, and the distance between nodes on the screen is controlled by the *spacing* setting. In addition, specific proteins can be highlighted, and the color scheme can be adjusted *via* the “Settings” button. Left-clicking nodes open a summary pop-up window, while right-clicking somewhere on the screen triggers a context menu. The latter features options to export the displayed interaction network as an image or to download the corresponding list of interactions. In the top left corner, an info box displays the currently viewed gene, the radius of the network, and the proportion of proteins contained in the network.

**FIGURE 5:**
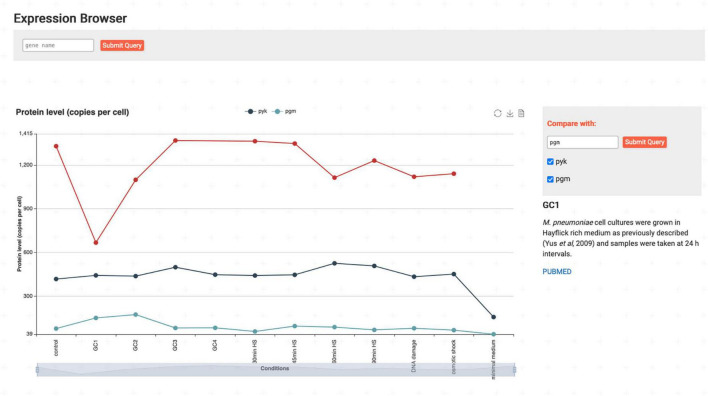
The *Expression Browser* shows protein levels and transcript levels (not included in the screenshot) for genes under various conditions. Data on additional genes can be loaded *via* the search bar for comparison. Here, protein levels for Eno are compared with Pyk and Pgm.

With the *Expression Browser* (Figure 5), the user can investigate protein and transcript levels (not shown) of genes/proteins under different conditions (Yus et al., 2009; Maier et al., 2011). Additional genes can be dynamically loaded for comparison using the search bar, and descriptions of the individual conditions are available by clicking on the corresponding data points. Options for data export are provided as well.

### Structure viewer

**FIGURE 6:**
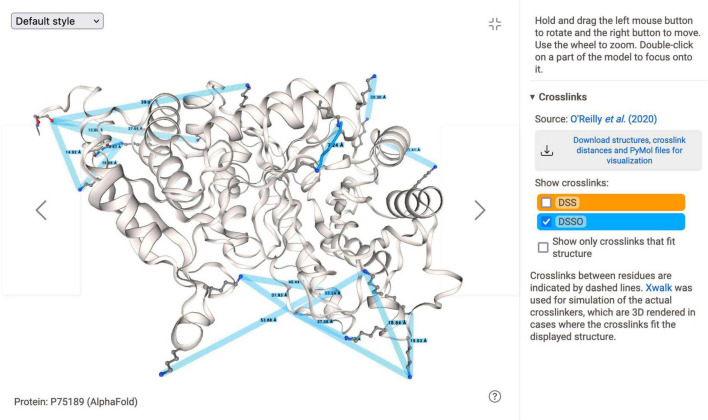
Fullscreen view of the *Structure Viewer*, which can be found on the sidebar of gene pages. It displays interactive 3D representations of available protein structures loaded from PDB or AlphaFold DB, and can also feature visualizations of internal cross-links. Using the control panel to the side of the view canvas, the visibility of cross-links can be toggled. The screenshot shows the AlphaFold structure prediction for *eno* and corresponding cross-links.

*Myco*Wiki introduces a new 3D protein structure viewer (Figure 6), which is not yet present in either *Subti*Wiki or *Syn*Wiki. It is able to load and display structures from the Protein Data Bank (PDB) (Burley et al., 2019) and structure predictions from the AlphaFold Protein Structure Database (Varadi et al., 2022). In addition, it features the visualization of internal cross-links based on data from a global *in vivo* study of protein-protein interactions (O’Reilly et al., 2020).

A minimized form of the *Structure Viewer* can be found on the gene page sidebar. While it features full functionality, a full-screen view is also available (shown in the figure), which includes information on how to control the viewer. By using the arrow icons, the user can cycle through the available structures of a protein, which are also found in the main body of the gene page in the section “The protein > Structure.” An info text in the bottom left corner indicates the currently viewed structure, and also links to the respective PDB or AlphaFold DB page. The user can choose different molecular representation styles from the drop-down selection in the upper left corner, such as renderings of the protein surface indicating hydrophobicity or electrostatic values. The visualization of structures and cross-links is performed with NGL Viewer, a web-based tool for molecular 3D graphics (Rose and Hildebrand, 2015). To the side of the viewer, additional information is displayed, including instructions about how to control the viewer and further details about the structure, if available.

As shown in the figure, visualization of cross-linking data is also available in *Myco*Wiki. The data result from a large-scale *in vivo* study (O’Reilly et al., 2020), in which whole-cell cross-linking mass spectrometry with two different cross-linkers (DSS and DSSO) was performed. For the *Structure Viewer*, only internal (intraprotein) cross-links were extracted and mapped to the AlphaFold structure predictions of the corresponding proteins. Of the 686 predictions assigned to *Myco*Wiki genes, internal cross-links were available for 441 structures. Cross-linked residues of a protein are highlighted in the viewer by dashed lines indicating the Euclidean distance between them. Furthermore, if the distance between the cross-linked residues is smaller than the spacer arms of the respective cross-linker, a molecular 3D representation of the linker is fitted to the structure. This representation was calculated and rendered using the program Xwalk, which determines the *Solvent Accessible Surface Distance* (SASD) between cross-linked amino acids (Kahraman et al., 2011). It corresponds to the shortest path between them only using solvent-occupied space, without passing through the protein surface. For DSS and DSSO, distances of 11.4 and 10.1 Å, respectively, plus a 1.5 Å tolerance were chosen as the maximum distance for which they could be fitted to the structure. In the *Structure Viewer*, the visibility of the different distance representations can be toggled *via* controls at the side of the viewer panel. In addition, a download link for an archive file of all structures and cross-link data is provided.

## Implementation and data

The *Myco*Wiki platform shares its framework with its predecessor *Subti*Wiki (Pedreira et al., 2022b). Accordingly, it is implemented using the same custom PHP backend framework and frontend functionality, and uses MySQL for its relational database. The application is hosted with Apache HTTP Server. Some differences *Subti*Wiki and *Syn*Wiki exist in presentation due to differing availability of data for the corresponding organisms, and some frontend features slightly vary in design.

*Myco*Wiki contains a mixture of manually curated information, which is gathered from recent publications and evaluated by experts, and individual bulk data imports from existing data sources, such as other databases or published experimental data. The platform received a lot of its original annotation from the database MyMpn (Wodke et al., 2015), which was discontinued in 2020. With its structure similar to the one of *Myco*Wiki, many parts of *MyMpn* could be directly adopted, such as genomic coordinates, enzyme commission numbers, and post-translational modifications. The main body of information on protein-protein interactions comes from a global *in vivo* study (O’Reilly et al., 2020).

Similar to *Subti*Wiki and *Syn*Wiki, a specialized set of categories was conceived for *Myco*Wiki. They are represented by a tree-like structure that classifies genes by their functions, but also groups them according to their localization, essentiality, or quality of characterization, among others. Table 1 shows the five top-level categories and their immediate subcategories, while lower-level subcategories are omitted. The main part of the categories has been adapted from *Syn*Wiki, however, “Virulence and pathogenicity” has been added as a top-level category to help characterization of the organism as a pathogen.

**TABLE 1:** List of top-level categories and their subcategories used in *Myco*Wiki, and the number of genes assigned to each of them.

	Subcategory	Number of genes
Cellular processes	Cell envelope and cell division	2
	Homeostasis	14
	Transporters	64
	Movement and adhesion	31
Metabolism	Amino acid acquisition and metabolism	18
	ATP synthesis	11
	Carbon metabolism	47
	Cofactor acquisition	14
	Sulfur metabolism	2
	Lipid metabolism	18
	Nucleotide metabolism	21
	Phosphate metabolism	5
	Detoxification reactions	6
Information processing	Genetics	60
	RNA synthesis and degradation	23
	Protein synthesis, modification and degradation	188
	Regulation	11
Virulence and pathogenicity	Virulence and pathogenicity	4
Groups of genes	Membrane proteins	188
	Secreted proteins	1
	Essential genes	332
	Conditional essential genes	58
	Universally conserved proteins	1
	Poorly characterized enzymes	44
	Proteins of unknown function	195

**FIGURE 7:**
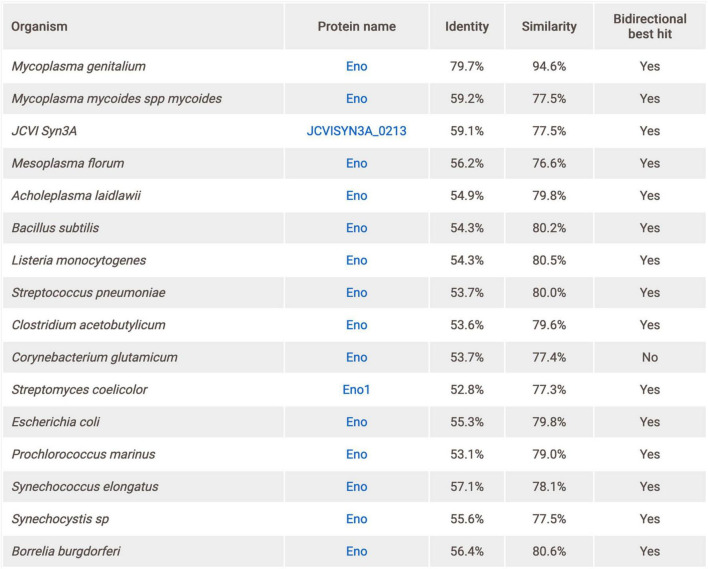
Protein homology table for Eno. Best BLAST hits for 16 representative related organisms are featured, and scores on identity and similarity are provided.

As in *Subti*Wiki and *Syn*Wiki, a list of precomputed best-hit protein homologs in selected related bacteria based on a FASTA pipeline (Pedreira et al., 2022b) has been added to each protein. A specialized page with a corresponding table (Figure 7) can be accessed from the “Homologs” section on any gene page. In total, 16 species were deemed representative of protein homologies, among them *Mycoplasma genitalium*, *Mycoplasma mycoides subsp. mycoides*, the artificial synthetic organism *M. mycoides JCVI Syn3A, Escherichia coli*, and *Bacillus subtilis*. Identity and similarity scores are given for each potential homolog, and an indication as to whether the homolog in question is also the best hit for the protein in the other direction.

To keep functional genome annotation up to date, joint efforts of the corresponding scientific community are required. Therefore, *Myco*Wiki is open to contributions from all scientists in the field of *Mycoplasma* research. This is a major distinctive feature as compared to other databases that include information on *M. pneumoniae*. In addition, MyMpn, as mentioned above, has not received updates in the past years. BioCyc, a large suite of databases for many species including *M. pneumoniae* (Karp et al., 2019), is only available behind a paywall after very few pages access whereas *Myco*Wiki is freely accessible to the scientific community. Finally, PATRIC, the Pathosystems Resource Integration Center (Davis et al., 2020), has a strong focus on genes rather than proteins. Thus, we are confident that *Myco*Wiki will become a valuable tool for the *Mycoplasma* research community.

## Curation and community

To further enhance the information provided in *Myco*Wiki, the *Mycoplasma* research community is invited to register and participate in the curation of the database. While access to the complete contents is free for everyone, only registered users are able to log in and contribute. The entries will be curated by the team behind *Myco*Wiki to ensure continuous high quality of the information provided.

## Future perspectives

With *Myco*Wiki, we have released a new comprehensive model organism database for the minimal bacterium *M. pneumoniae*. It utilizes the popular framework of *Subti*Wiki to facilitate intuitive exploration of the available annotation. Particular focus is put on the interactions between different genes and proteins, which may support the scientific community in the development of novel research hypotheses. Customized categories are used to classify the functions and other qualities of genes and their products concisely. In addition, the inclusion of a homology analysis could help to infer the functional annotation of genes. AlphaFold structure predictions have been assigned for the proteins, allowing a visual representation even in cases where no experimentally determined structure exists. While the rendering of internal cross-links for these structures can give an idea about the quality of the prediction, interprotein cross-links could be integrated in the future to explore the interaction between proteins in more detail. We hope that *Myco*Wiki will become a valuable tool for the *M. pneumoniae* research community, and in turn an asset to the ongoing systems and synthetic biology research. Moreover, the wealth of information provided in *Myco*Wiki and the easy access to classes of proteins based on the categories will help in the further development of *M. pneumoniae* as a chassis to target therapeutical compounds.

For the family of databases that includes *Myco*Wiki, *Subti*Wiki, and *Syn*Wiki, we will develop novel features including protein-RNA, RNA-RNA, and protein-metabolite interactions that will certainly enhance the value of the databases.

## Data availability statement

The original contributions presented in this study are included in the article/supplementary material, further inquiries can be directed to the corresponding author.

## Author contributions

CE, BZ, and TP developed the framework of the database and integrated the data. BH performed initial work for the development of the structure viewer and integration of cross-link data. ML-S and LS provided data for the database. JS provided funding and supervised the development of the database. CE and JS wrote the manuscript. All authors read and approved the current submission.
